# Response of *Amaranthus* spp. following exposure to sublethal herbicide rates via spray particle drift

**DOI:** 10.1371/journal.pone.0220014

**Published:** 2019-07-18

**Authors:** Bruno C. Vieira, Joe D. Luck, Keenan L. Amundsen, Todd A. Gaines, Rodrigo Werle, Greg R. Kruger

**Affiliations:** 1 West Central Research and Extension Center, University of Nebraska-Lincoln, North Platte, NE, United States of America; 2 Department of Biological Systems Engineering, University of Nebraska-Lincoln, Lincoln, NE, United States of America; 3 Department of Agronomy and Horticulture, University of Nebraska-Lincoln, Lincoln, NE, United States of America; 4 Department of Bioagricultural Sciences and Pest Management, Colorado State University, Fort Collins, CO, United States of America; 5 Department of Agronomy, University of Wisconsin–Madison, Madison, WI, United States of America; California State University Fresno, UNITED STATES

## Abstract

The adverse consequences of herbicide drift towards sensitive crops have been extensively reported in the literature. However, little to no information is available on the consequences of herbicide drift onto weed species inhabiting boundaries of agricultural fields. Exposure to herbicide drift could be detrimental to long-term weed management as several weed species have evolved herbicide-resistance after recurrent selection with sublethal herbicide rates This study investigated the deposition of glyphosate, 2,4-D, and dicamba spray particle drift from applications with two different nozzles in a low speed wind tunnel, and their impact on growth and development of *Amaranthus* spp. Herbicide drift resulted in biomass reduction or complete plant mortality. Inflection points (distance to 50% biomass reduction) for *Amaranthus tuberculatus* were 7.7, 4.0, and 4.1 m downwind distance for glyphosate, 2,4-D, and dicamba applications with the flat-fan nozzle, respectively, whereas these values corresponded to 2.8, 2.5, and 1.9 m for applications with the air-inclusion nozzle. Inflection points for *Amaranthus palmeri* biomass reduction were 16.3, 10.9, and 11.5 m for glyphosate, 2,4-D, and dicamba applications with the flat-fan nozzle, respectively, whereas these values corresponded to 7.6, 5.4, and 5.4 m for applications with the air-inclusion nozzle. Plants were more sensitive to glyphosate at higher exposure rates than other herbicides, whereas plants were more sensitive to 2,4-D and dicamba at lower exposure rates compared to glyphosate. Applications with the flat-fan nozzle resulted in 32.3 and 11.5% drift of the applied rate at 1.0 and 3.0 m downwind, respectively, whereas the air-inclusion nozzle decreased the dose exposure in the same distances (11.4 and 2.7%, respectively). Herbicide drift towards field boundaries was influenced by nozzle design and exposed weeds to herbicide rates previously reported to select for herbicide-resistant biotypes.

## Introduction

Spray drift is defined as the part of the application (particles or vapors) that is deflected away from the target during or following applications [1]. Many environmental and application technique factors influence spray particle drift, such as wind speed and direction, sprayer boom height, and spray droplet size [2–4]. Spray droplet size which is directly influenced by nozzle design, nozzle orifice size, operating pressure, and physicochemical properties of the solution, is often the focal point of particle drift mitigation efforts [5–7].

**Fig 1:**
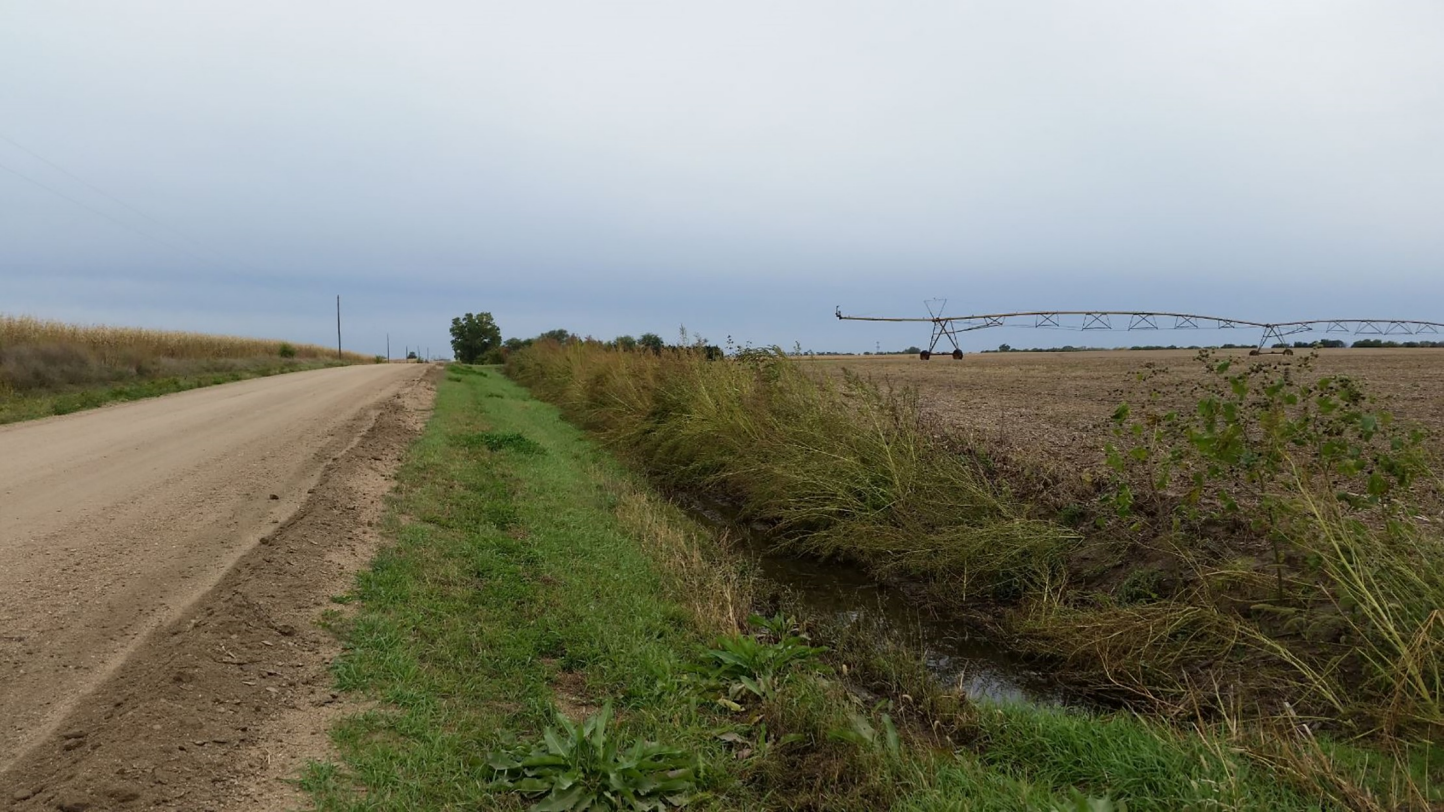
Waterhemp (*Amaranthus tuberculatus*) population located on field border in eastern Nebraska.

Risk assessment of herbicide drift includes the surrounding vegetation characterization, as non-target sensitive vegetation coexist with agricultural fields [8,9]. The adverse consequences of herbicide drift towards sensitive crops have been extensively reported in the literature [10–13]. However, little to no information is available on the consequences of herbicide drift on agricultural weed species. Weed species including horseweed (*Erigeron canadensis* L.), waterhemp [*Amaranthus tuberculatus* (Moq.) J. D. Sauer], Palmer amaranth (*Amaranthus palmeri* S. Wats.), velvetleaf (*Abutilon theophrasti* Medik), giant ragweed (*Ambrosia trifida* L. AMBTR), and others are often abundant in field boundaries and ditches surrounding agricultural lands in the US Midwest [14–17] (Fig 1).

Exposure to herbicide drift could be detrimental to long-term weed management as several weed species have evolved resistance after recurrent selection with sublethal herbicide rates [18–27]. Previous research reported that recurrent selection with low rates of herbicides progressively selected for herbicide metabolism alleles present within the standing genetic variation of the population, additively leading to herbicide resistance [28–30]. In most recurrent selection studies, weed populations selected with sublethal rates of a given herbicide also evolved resistance to other herbicide sites of action [18,22,23,26]. This highlights the nature of non-target site resistance (NTSR) and influence of metabolic alleles selected in weed populations upon recurrent selection with low herbicides rates [29,31,32]. It has been suggested that recurrent selection with sublethal doses of herbicides not only select polygenic alleles within the standing genetic variation of the population, but also could induce new stress-related mutations within surviving individuals [33]. Furthermore, it has been suggested that sublethal herbicide rates could act as stress agents inducing DNA mutations, epigenetic alterations, transcriptional remodeling, protein modifications, and other events that could ultimately confer levels of herbicide resistance [34]. Stress-induced epigenetic changes (DNA methylation, histone modifications, and others) are normally reverted soon after stress exposure, although in specific cases they can be carried over for multiple generations [35]. The reproductive system of weed species influences herbicide resistance evolution. For instance, when plants are recurrently selected with sublethal rates of herbicides, recombination and accumulation of minor resistance genes can occur at a faster rate in cross-pollinated species such as waterhemp and Palmer amaranth [20,36].

Despite the potential adverse implications towards resistance evolution from sublethal rate exposure via herbicide drift, near-field weed populations are often ignored and not managed in agricultural landscapes [14,15,17,37]. Therefore, the objectives of this study were to investigate the near-field deposition of glyphosate, 2,4-D, and dicamba spray particle drift from applications with two different nozzles (different droplet spectrum resulting in low and high drift potentials) in a low speed wind tunnel, and their impact on waterhemp and Palmer amaranth growth and development under controlled environment.

## Material and methods

### Plant material

A waterhemp population collected from a corn field (*Zea mays* L.) in northeastern Nebraska (Cuming County) in the fall of 2014, and a Palmer amaranth population collected from a sorghum (*Sorghum bicolor* L.) field in southwestern Nebraska (Hayes County) in the fall of 2015 were used in this study. No specific permissions were required for field seed collections, and field collections did not involve endangered or protected species. Both waterhemp and Palmer amaranth populations were previously confirmed susceptible to glyphosate, 2,4-D, and dicamba with dose-response bioassays (unpublished data). Waterhemp and Palmer amaranth seeds were sown into plastic tubes (1 L) containing commercial potting mix (Berger BM7 Bark Mix, Saint Modeste, QC, Canada) and maintained under greenhouse conditions (30/20 C [day/night] with a 16 h photoperiod) at the Pesticide Application Technology Laboratory (University of Nebraska-Lincoln, West Central Research and Extension Center, North Platte, NE). LED growth lights (520 μmol s^−1^, Philips Lighting, Somerset, NJ, USA) provided supplemental lighting to ensure a 16-h photoperiod. Plants were supplied with water including fertilizer solution (0.2% v/v) as needed (UNL 5-1-4, Wilbur-Ellis Agribusiness, Aurora, CO, USA).

### Droplet size study

A droplet size study was conducted in the low speed wind tunnel at the Pesticide Application Technology Laboratory. Droplet size distribution data were collected using a Sympatec Helos/Vario KR laser diffraction system (Sympatec Inc., Clausthal, Germany) measuring at a distance of 0.3 m from the nozzle tip. The diffraction system was equipped with a R7 lens which detects droplets ranging from 9 to 3700 μm in diameter. Nozzles were attached to an actuator and traversed vertically at constant speed (0.2 m s^-1^) to ensure the entire spray plume crossed the laser diffraction system [38]. Applications were performed with two even (banding) nozzles; a conventional flat-fan nozzle (TP95015EVS) and an air-inclusion (AI) nozzle (AI95015EVS) (TeeJet Technologies Spraying Systems Co., Glendale Heights, IL, USA); and three herbicide solutions: glyphosate, 2,4-D, and dicamba (Table 1). The glyphosate treatment had the addition of ammonium sulfate solution at 5% v/v to overcome antagonistic effects of cationic salts in hard water (Bronc^®^, Wilbur-Ellis Agribusiness, Aurora, CO, USA). Solutions were prepared at 140 L ha^-1^ carrier volume. Applications were performed at 230 kPa with constant wind speed of 6.71 m s^-1^. The DV_0.1_, DV_0.5_, and DV_0.9_ (droplet diameters which 10, 50, and 90% of the spray volume are contained in droplets of smaller diameter, respectively), and the percentage of the spray volume in droplets smaller than 150 μm (driftable fines) were recorded. The relative span (RS), a dimensionless parameter that estimates the distribution spread and its homogeneity was calculated [39] (Eq 1):

$$\frac{\left({DV}_{0.9}-{DV}_{0.1}\right)}{{DV}_{0.5}}$$ [1]

**Table 1:** Herbicide solutions, rates, and product manufacturers for solutions tested in the droplet size and spray particle drift studies^a^.

Herbicide	Active ingredient	Product manufacturer	Rate
Clarity^®^	Dicamba diglycolamine salt	BASF Corporation, Research, Triangle Park, NC, USA	280 g ae ha^-1^
Roundup PowerMax^®^	Glyphosate potassium salt	Bayer CropScience, Research, Triangle Park, NC, USA	867 g ae ha^-1^
Weedar^®^ 64	2,4-D dimethylamine salt	Nufarm Inc, Alsip, IL, USA	532 g ae ha^-1^

^a^Glyphosate solution had the addition of ammonium sulfate solution at 5% v/v (Bronc^®^, Wilbur-Ellis Agribusiness, Aurora, CO, USA).

The treatment design was a factorial arrangement with herbicide solution and nozzle as factors in a complete randomized experimental design with three replications and repeated. Droplet size data were subjected to analysis of variance in SAS (SAS v9.4, SAS Institute Inc., Cary, NC, USA) and comparisons among treatments were performed using Fisher’s Protected LSD test (P ≤ 0.05).

### Wind tunnel particle drift study

**Fig 2:**
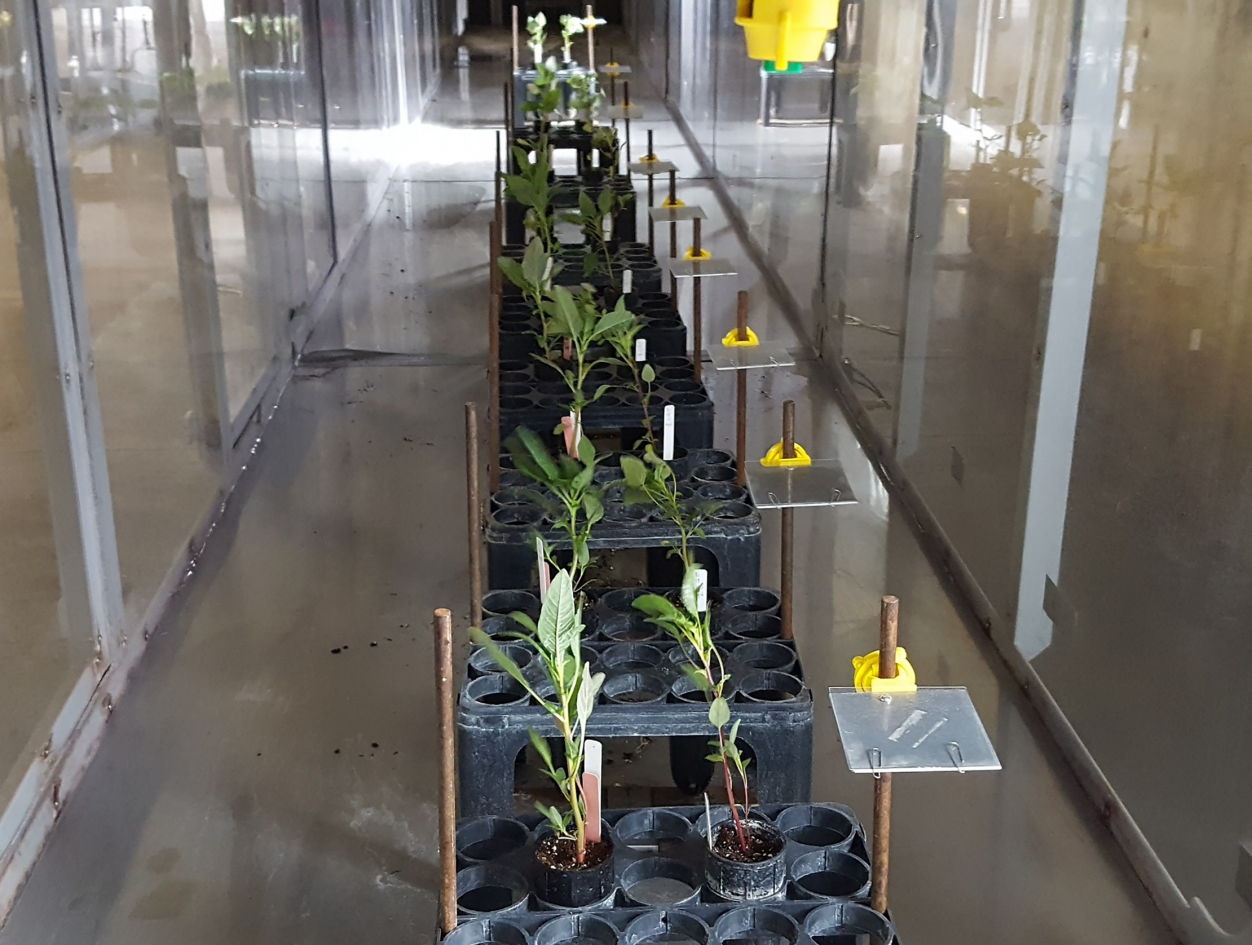
Herbicide particle drift study conducted in the low speed wind tunnel with waterhemp (*Amaranthus tuberculatus*), Palmer amaranth (*Amaranthus palmeri*), and drift collectors (Mylar cards) positioned at different downwind distances from the nozzle.

A spray particle drift deposition study was conducted in the low speed wind tunnel at the Pesticide Application Technology Laboratory. Glyphosate, 2,4-D, and dicamba solutions were prepared as previously described (Table 1) with the addition of 1,3,6,8-pyrene tetra sulfonic acid tetra sodium salt (PTSA) as a fluorescent tracer (Spectra Colors Corporation, Kearny, NJ, USA) at 1000 ppm concentration [40]. Herbicide solutions were sprayed at 140 L ha^-1^ using two different even nozzles (banding) at 230 kPa (AI95015EVS and TP95015EVS) under a 4.47 m s^-1^ wind speed. The average air temperature and relative humidity during this study were 25 C and 45%, respectively. Mylar cards (100 mm x 100 mm) (Grafix Plastics, Cleveland, OH) were used to collect particle drift deposition at different downwind distances: 1.0, 1.5, 2.0, 2.5, 3.0, 4.0, 5.0, 7.0, and 12.0 m from the nozzle. Simultaneously, waterhemp and Palmer amaranth plants (15–20 cm-tall) were also positioned at the same downwind distances (Fig 2). Applications were performed at 51 cm height in relation to Mylar cards and plants.

After applications, Mylar cards were collected and placed into pre-labeled plastic zip-top bags and were immediately transferred to a dark container to avoid PTSA photodegradation. Spray particle drift deposition was determined for each Mylar card by fluorometric analysis at the Pesticide Application Technology Laboratory. Mylar cards were washed using 40 ml of a 9:1 solution of distilled water and 91% isopropyl alcohol. With the tracer completely suspended, a 1.5 ml aliquot was transferred to glass cuvette and analyzed using a Trilogy® fluorimeter with a PTSA module (Turner Designs, Sunnyvale, CA, USA). Relative fluorescence units (RFU) data were converted into mg L^-1^ using a calibration curve for the tracer, and posteriorly to deposition percentage as compared to the theoretical application rate of 140 L ha^-1^. The deposition data for each nozzle by herbicide solution combination (nozzle*herbicide) was estimated with a four-parameter symmetric log-logistic model using the *drc* package in R software (R Foundation for Statistical Computing, Vienna, Austria) (Eq 2):

y=c+(d–c/1+exp(b(logx–loge))) [2]

where *y* represents deposition (% from applied rate), *b* is the slope at the inflection point, *c* is the lower limit of the model (fixed to 0%), *d* is the upper limit (applied rate fixed to 100%), and *e* is the inflection point (distance to 50% spray drift deposition) [41]. The distance to 5% application rate deposition (*D*_*5*_) was estimated for each nozzle*herbicide combination.

After applications, waterhemp and Palmer Amaranth plants were maintained under greenhouse conditions as previously described. Above ground plant biomass was harvested 28 days after treatment (DAT) and oven dried at 65°C to constant weight. The biomass data were converted into percentage of biomass reduction as compared to the untreated control. The symmetric four-parameter log-logistic model was used to describe biomass reduction using the drc package in R statistical software (Eq 2), where *y* represents biomass reduction (%), *b* is the slope at the inflection point, *c* is the lower limit of the model (fixed to 0%), *d* is the upper limit, and *e* is the inflection point (distance to 50% biomass reduction).

In swath (0 m distance) plant biomass reduction for each nozzle*herbicide treatment was estimated with herbicide applications using a research spray chamber calibrated to deliver 140 L ha^-1^ with the same nozzles, herbicide solutions, and spraying parameters used in the wind tunnel study.

## Results and discussion

### Droplet size

A significant interaction between nozzle design and herbicide solution was detected for the DV_0.1_ (p = 0.0002), DV_0.5_ (p < 0.0001), DV_0.9_ (p < 0.0001), RS (p < 0.0001), and driftable fines (p < 0.0001). Nozzle design had the greatest influence on droplet size, whereas herbicide solution had minor impact as previously reported [5,42,43] (Table 2). The preorifice component of the AI nozzle is designed to reduce the solution pressure as it exits the nozzle, thereby increasing the droplet size of the spray [5,42].

**Table 2:** Droplet size distribution and spray classification for the two nozzles and three herbicide solutions tested in the droplet size and spray particle drift study at 230 kPa^a^.

Nozzle^b^	Herbicide^c^	Droplet size characteristics^d^					Spray classification^e^
		DV_0.1_	DV_0.5_	DV_0.9_	RS	Driftable fines	
		^______________^ μm ^______________^				%	
TP95015EVS	Glyphosate	89 D	201 D	348 E	1.29 A	30.7 A	F
	2,4-D	98 C	212 C	360 D	1.23 B	26.2 C	F
	Dicamba	96 C	209 C	355 DE	1.24 B	26.9 B	F
AI95015EVS	Glyphosate	392 B	805 A	1212 B	1.02 C	0.6 D	UC
	2,4-D	408 A	801 A	1223 A	1.02 C	0.4 D	UC
	Dicamba	411 A	789 B	1166 C	0.96 D	0.4 D	UC

^a^Means within a column followed by the same letter are not significantly different based on the LSD test (P ≤ 0.05).

^b^TeeJet Technologies, Spraying Systems Co., Glendale Heights, IL, USA.

^c^Glyphosate solution had the addition of ammonium sulfate solution at 5% v/v (Bronc^®^, Wilbur-Ellis Agribusiness, Aurora, CO, USA).

^d^Abbreviations: DV_0.1_, DV_0.5_, and DV_0.9_: Parameters which represent the droplet size such that 10, 50, and 90% of the spray volume is contained in droplets of lesser values, respectively

Driftable fines: Percent of spray volume that contains droplets less than 150 μm diameter

RS: Relative span, a dimensionless parameter that estimates the spread of a distribution.

^e^The spray classifications for this study were based on reference curves created from reference nozzle data at the Pesticide Application Technology Laboratory as described by ASABE S572.1 where F = Fine, and UC = Ultra Coarse.

### Wind tunnel particle drift deposition

**Fig 3:**
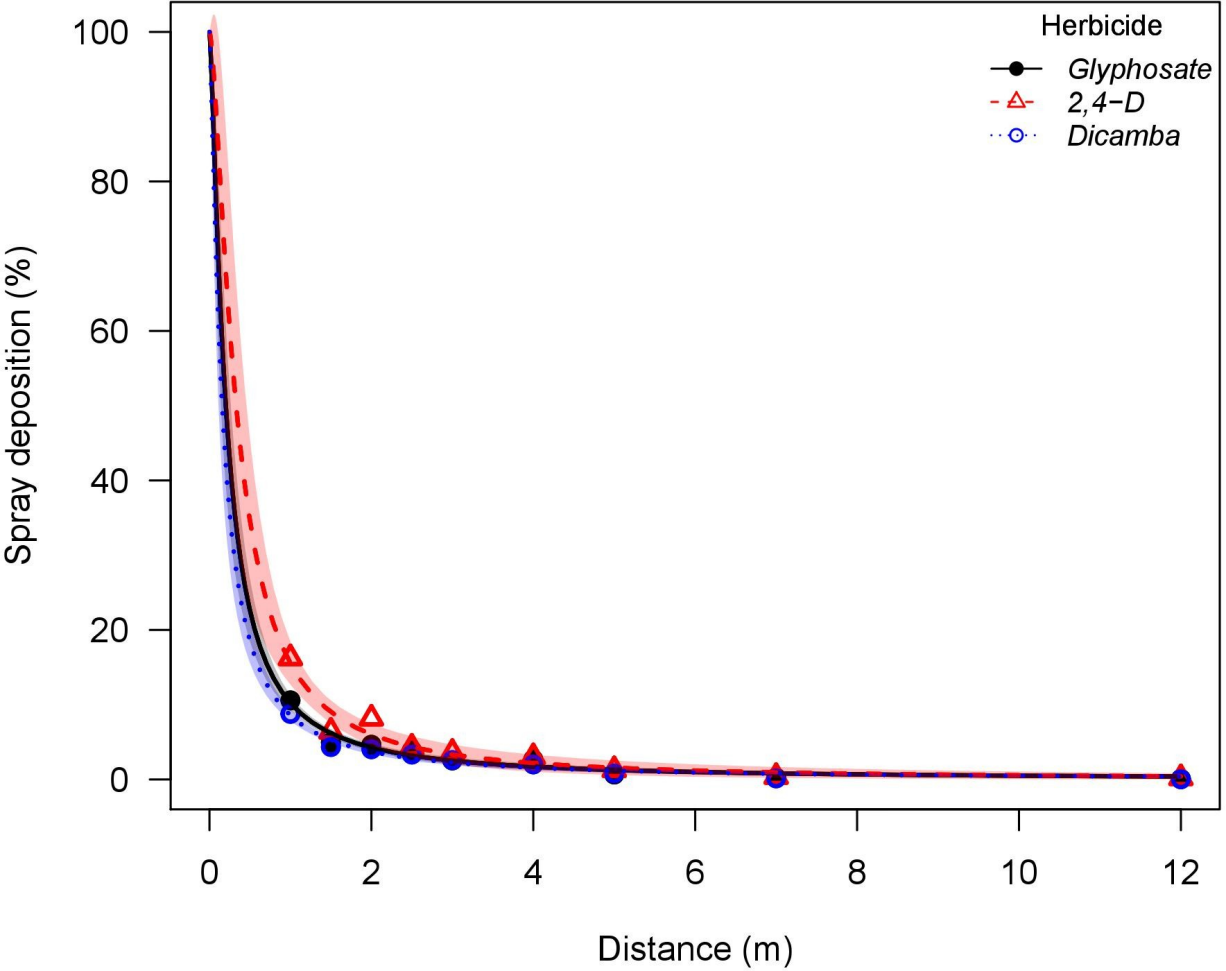
Glyphosate, 2,4-D, and dicamba particle drift study using an air-inclusion nozzle (AI95015EVS) conducted in a low speed wind tunnel. Shaded area indicates the 95% confidence limits. Glyphosate solution had the addition of ammonium sulfate solution at 5% v/v (Bronc^®^, Wilbur-Ellis Agribusiness, Aurora, CO, USA).

**Fig 4:**
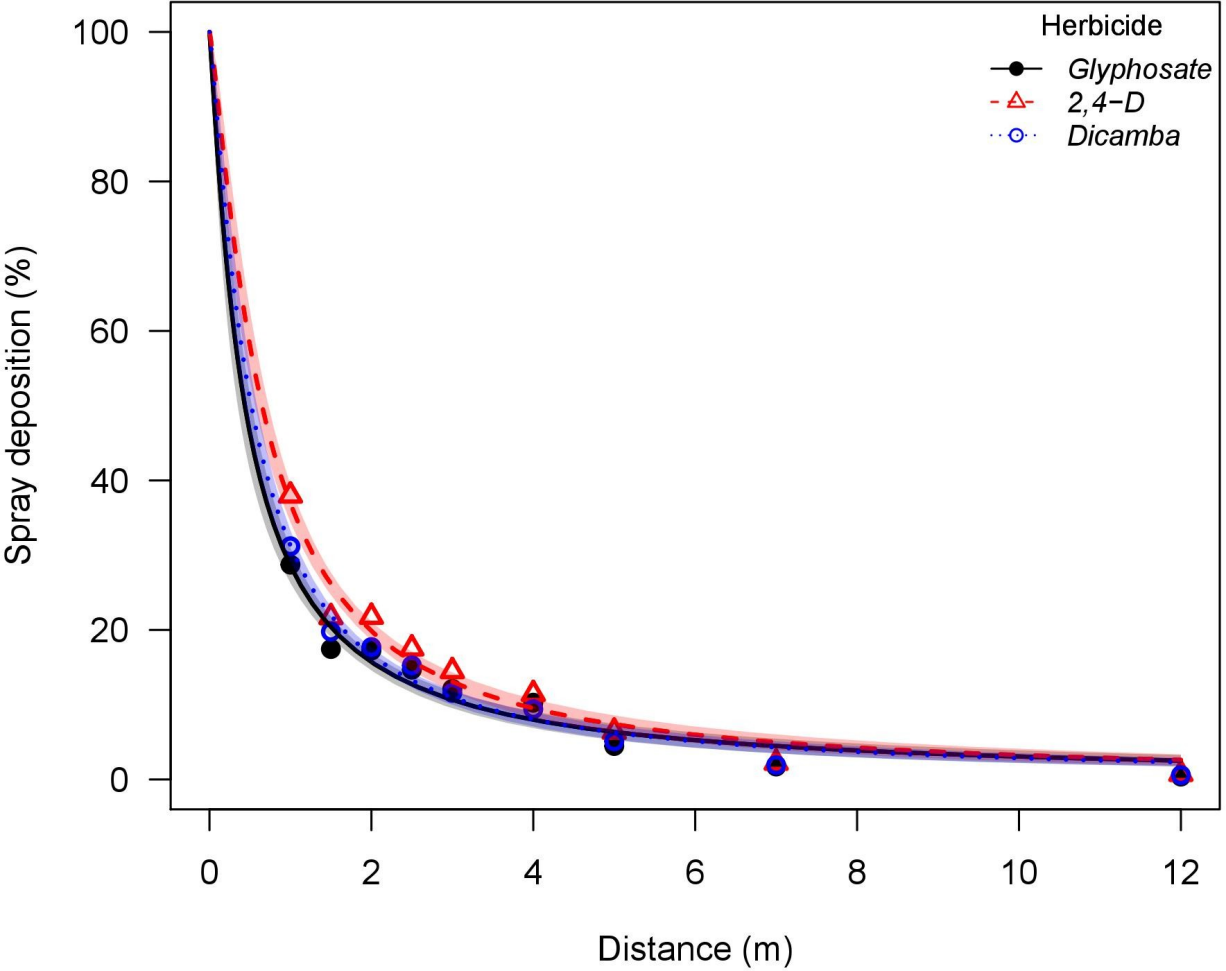
Glyphosate, 2,4-D, and dicamba particle drift study using a flat-fan nozzle (TP95015EVS) conducted at a low speed wind tunnel. Shaded area indicates the 95% confidence limits. Glyphosate solution had the addition of ammonium sulfate solution at 5% v/v (Bronc^®^, Wilbur-Ellis Agribusiness, Aurora, CO, USA).

The nozzle treatments selected herein created two scenarios: a low drift potential (AI nozzle producing Ultra Coarse droplets with less than 1% of driftable fines) and a high drift potential (flat-fan nozzle producing Fine droplets with more than 25% of driftable fines). The estimated particle drift potential of treatments included in this wind tunnel study are consistent with previously reported field scale particle drift potential, where similar nozzle designs, droplet size classifications, and study methods were used. A study reported that 5% of applications of water with PTSA solution (93.5 L ha^-1^) using an AI nozzle at an average wind speed of 5.7 m s^-1^ deposited at 2.3 m downwind, whereas this distance corresponded to 4.5 m for applications with a flat-fan nozzle [43]. In this wind tunnel study, applications with the AI nozzle had 5% of the applied rate being deposited at 1.9 m downwind when herbicides were pooled, whereas this distance corresponded to 6.5 m for applications with the flat-fan nozzle. This indicates that the wind tunnel drift simulation method reproduced near-field spray drift conditions (Figs 3 and 4). Herbicide applications with the AI nozzle had smaller *e* parameter (distance to 50% spray drift deposition), ranging from 0.16 to 0.33 m across herbicides, when compared to applications with the flat-fan nozzle (0.44 to 0.65 m) (Table 3). The same trend was observed in the *D*_*5*_ parameter, where applications with the AI nozzle had 5% of the total applied rate being deposited from 1.57 to 2.27 m across herbicides, whereas these distances are increased to 6.11 and 6.97 m with the flat-fan nozzle. These results indicate the greater spray particle drift potential of the flat-fan nozzle. The greater *b* parameter (slope at the inflection point) of applications with the AI nozzle (ranging from 1.28 to 1.52 across herbicides) when compared to the flat-fan nozzle (1.10 to 1.24) indicates a faster decay rate of spray deposits resulting in less spray deposition at further downwind distances.

**Table 3:** Log-logistic model parameters estimates, standard errors, and distance to 5% application rate deposition (*D*_*5*_) as influenced by downwind distance for each nozzle*herbicide treatment combination tested in the spray particle drift study^a^.

Nozzle^b^	Herbicide	Log-logistic model parameters^c^		
		*b*	*e*	*D*_*5*_
			^_______________^ m ^_______________^	
TP95015EVS	Glyphosate	1.10 ± 0.07	0.44 ± 0.04	6.28 ± 0.60
	2,4-D	1.24 ± 0.07	0.65 ± 0.04	6.97 ± 0.56
	Dicamba	1.19 ± 0.07	0.52 ± 0.04	6.11 ± 0.53
AI95015EVS	Glyphosate	1.36 ± 0.19	0.20 ± 0.05	1.77 ± 0.12
	2,4-D	1.52 ± 0.15	0.33 ± 0.05	2.27 ± 0.14
	Dicamba	1.28 ± 0.21	0.16 ± 0.06	1.57 ± 0.11

^a^Glyphosate solution had the addition of ammonium sulfate solution at 5% v/v (Bronc^®^, Wilbur-Ellis Agribusiness, Aurora, CO, USA).

^b^TeeJet Technologies, Spraying Systems Co., Glendale Heights, IL.

^*c*^*b* parameter corresponds to the slope at the inflection point; *e* parameter corresponds to the distance to 50% application deposition; *c* parameter (lower limit) fixed to 0%; *d* parameter (upper limit) fixed to 100%; *D*_*5*_ corresponds to the distance to 5% application rate deposition.

These findings corroborate the results from a field study investigating spray particle drift [44], where applications (water plus fluorescent tracer) with AI nozzles resulted in less particle drift compared to applications with conventional flat-fan nozzles. It has been reported that the distance where sorghum plants were lethally injured by glyphosate drift decreased 34% for applications with AI nozzles compared to conventional flat-fan nozzles [45]. Similar wind tunnel study results were reported, where applications of dicamba alone and in tank mixtures with glyphosate using AI nozzles resulted in less herbicide particle drift compared to conventional flat-fan nozzles [46,47].

### Plants response to herbicide drift

Herbicide drift exposure subjected waterhemp and Palmer amaranth plants to either physiological stress (biomass reduction) or mortality (Table 4). The parameter estimates for the log-logistic biomass reduction model for waterhemp and Palmer amaranth are presented in Tables 5 and 6, respectively. The estimated *d* parameters (in-swath biomass reduction or upper limit) were greater than 84% biomass reduction for all nozzle*herbicide treatments, confirming that waterhemp and Palmer amaranth populations used in this study were susceptible to glyphosate, 2,4-D, and dicamba. Plants had greater biomass reduction when exposed to herbicide drift from applications with the flat-fan nozzle (greater drift potential).

**Table 4:** Waterhemp and Palmer amaranth mortality and estimations of biomass reduction using a log-logistic model as influenced by downwind distances for each nozzle*herbicide combination tested in the spray particle drift study^a^^b^.

Nozzle^c^	Distance	Waterhemp mortality (biomass reduction)			Palmer amaranth mortality (biomass reduction)		
TP95015EVS		Glyphosate	2,4-D	Dicamba	Glyphosate	2,4-D	Dicamba
	m	^_________________________________________^ % ^____________________________________^					
	1.0	100 (89)	83 (83)	83 (74)	100 (93)	0 (75)	67 (86)
	1.5	83 (87)	83 (75)	50 (67)	100 (93)	0 (72)	83 (83)
	2.0	17 (85)	0 (69)	17 (62)	100 (93)	0 (69)	17 (79)
	2.5	17 (82)	17 (63)	0 (57)	83 (92)	0 (66)	17 (77)
	3.0	0 (78)	0 (57)	0 (53)	83 (92)	0 (64)	0 (74)
	4.0	17 (71)	0 (49)	0 (46)	83 (91)	0 (60)	0 (69)
	5.0	0 (63)	0 (42)	0 (40)	67 (89)	0 (56)	0 (65)
	7.0	0 (50)	0 (32)	0 (33)	33 (83)	0 (50)	0 (58)
	12.0	0 (27)	0 (19)	0 (22)	0 (64)	0 (41)	0 (47)
AI95015EVS	1.0	100 (91)	0 (60)	17 (54)	100 (94)	0 (59)	0 (59)
	1.5	67 (80)	17 (53)	0 (49)	83 (91)	0 (55)	0 (56)
	2.0	0 (67)	0 (48)	0 (45)	100 (87)	0 (53)	0 (54)
	2.5	0 (55)	0 (44)	0 (42)	33 (83)	0 (50)	0 (52)
	3.0	0 (44)	0 (41)	0 (39)	33 (79)	0 (49)	0 (50)
	4.0	0 (28)	0 (35)	0 (35)	17 (71)	0 (46)	0 (48)
	5.0	0 (19)	0 (32)	0 (33)	0 (64)	0 (44)	0 (46)
	7.0	0 (9)	0 (26)	0 (28)	0 (52)	0 (40)	0 (43)
	12.0	0 (3)	0 (19)	0 (22)	0 (32)	0 (35)	0 (39)

^a^Glyphosate solution had the addition of ammonium sulfate solution at 5% v/v (Bronc^®^, Wilbur-Ellis Agribusiness, Aurora, CO, USA).

^b^Biomass reduction as compared to the untreated control.

^c^TeeJet Technologies, Spraying Systems Co., Glendale Heights, IL.

**Table 5:** Log-logistic model parameters estimates and standard errors for waterhemp biomass reduction as influenced by downwind distance for each nozzle*herbicide combinations tested in the spray particle drift study^a^^b^.

Nozzle^c^	Herbicide	Log-logistic model parameters^d^		
		*b*	*d* (%)	*e* (m)
TP95015EVS	Glyphosate	1.92 ± 0.38	91.04 ± 4.00	7.71 ± 0.69
	2,4-D	1.28 ± 0.17	96.79 ± 4.58	4.04 ± 0.45
	Dicamba	1.07 ± 0.16	90.32 ± 4.87	4.10 ± 0.59
AI95015EVS	Glyphosate	2.44 ± 0.33	98.18 ± 4.24	2.75 ± 0.16
	2,4-D	0.83 ± 0.14	87.96 ± 4.93	2.48 ± 0.43
	Dicamba	0.61 ± 0.12	90.80 ± 5.04	1.91 ± 0.46

^a^Glyphosate solution had the addition of ammonium sulfate solution at 5% v/v (Bronc^®^, Wilbur-Ellis Agribusiness, Aurora, CO, USA).

^b^Biomass reduction as compared to the untreated control.

^c^TeeJet Technologies, Spraying Systems Co., Glendale Heights, IL.

^d^*c* parameter (lower limit) fixed to 0%; *b* parameter corresponds to the slope at the inflection point; *d* parameter corresponds to the upper limit, *e* parameter corresponds to the distance to 50% biomass reduction.

**Table 6:** Log-logistic model parameters estimates and standard errors for Palmer amaranth biomass reduction as influenced by downwind distance for each nozzle*herbicide combinations tested in the spray particle drift study^a^^b^.

Nozzle^c^	Herbicide	Log-logistic model parameters^d^		
		*b*	*d* (%)	*e* (m)
TP95015EVS	Glyphosate	2.55 ± 0.83	93.13 ± 1.92	16.31 ± 2.24
	2,4-D	0.86 ± 0.14	84.76 ± 3.55	10.91 ± 1.98
	Dicamba	0.91 ± 0.14	95.59 ± 3.75	11.50 ± 1.82
AI95015EVS	Glyphosate	1.53 ± 0.19	98.28 ± 3.09	7.55 ± 0.63
	2,4-D	0.46 ± 0.10	85.85 ± 4.27	5.38 ± 1.53
	Dicamba	0.37 ± 0.09	90.98 ± 4.28	5.37 ± 1.80

^a^Glyphosate solution had the addition of ammonium sulfate solution at 5% v/v (Bronc^®^, Wilbur-Ellis Agribusiness, Aurora, CO, USA).

^b^Biomass reduction as compared to the untreated control.

^c^TeeJet Technologies, Spraying Systems Co., Glendale Heights, IL.

^d^*c* parameter (lower limit) fixed to 0%; *b* parameter corresponds to the slope at the inflection point; *d* parameter corresponds to the upper limit, *e* parameter corresponds to the distance to 50% biomass reduction; *D*_*5*_ corresponds to the distance with 5% application rate deposition.

**Fig 5:**
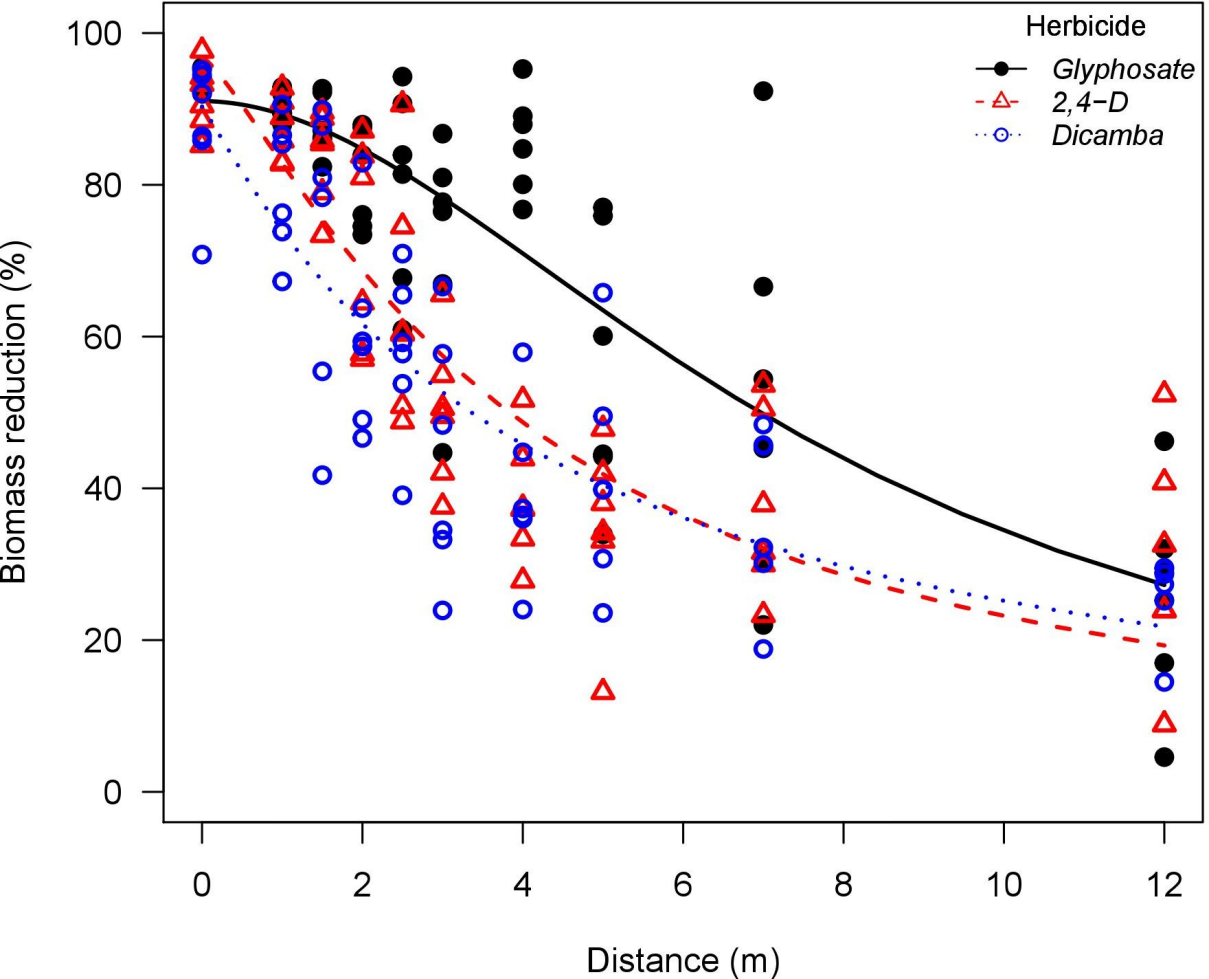
Waterhemp (*Amaranthus tuberculatus*) biomass reduction as influenced by glyphosate, 2,4-D, and dicamba particle drift using a flat-fan nozzle (TP95015EVS) in a low speed wind tunnel. Glyphosate solution had the addition of ammonium sulfate solution at 5% v/v (Bronc^®^, Wilbur-Ellis Agribusiness, Aurora, CO, USA).

**Fig 6:**
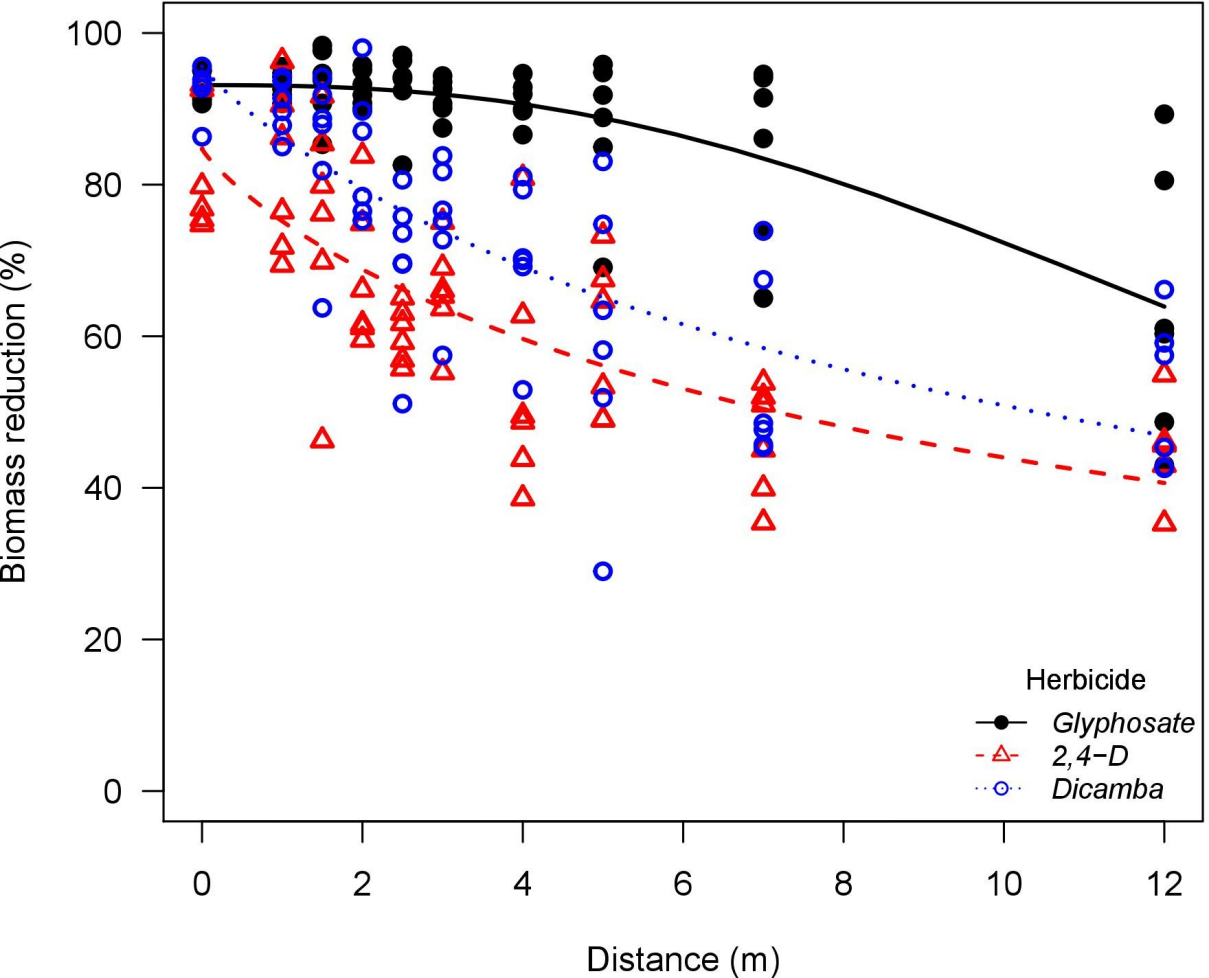
Palmer amaranth (*Amaranthus palmeri*) biomass reduction as influenced by glyphosate, 2,4-D, and dicamba particle drift using a flat-fan nozzle (TP95015EVS) in a low speed wind tunnel. Glyphosate solution had the addition of ammonium sulfate solution at 5% v/v (Bronc^®^, Wilbur-Ellis Agribusiness, Aurora, CO, USA).

Across the herbicides tested, Palmer amaranth had higher biomass reduction compared to waterhemp. The susceptibility differences between waterhemp and Palmer amaranth were more evident with glyphosate, corroborating a previous report [17]. Palmer amaranth was extremely susceptible to glyphosate drift from both nozzles, in which the biomass reduction curve as influenced by downwind distances did not even reach the *e* parameter (distance to 50% biomass reduction) for applications with the flat-fan nozzle (Figs 5 and 6). In scenarios where the weed biotypes are extremely susceptible to a given herbicide, selection pressure will take place in extended downwind distances from the sprayed area as further distance is required to plants reach the no observable effect level (NOEL).

**Fig 7:**
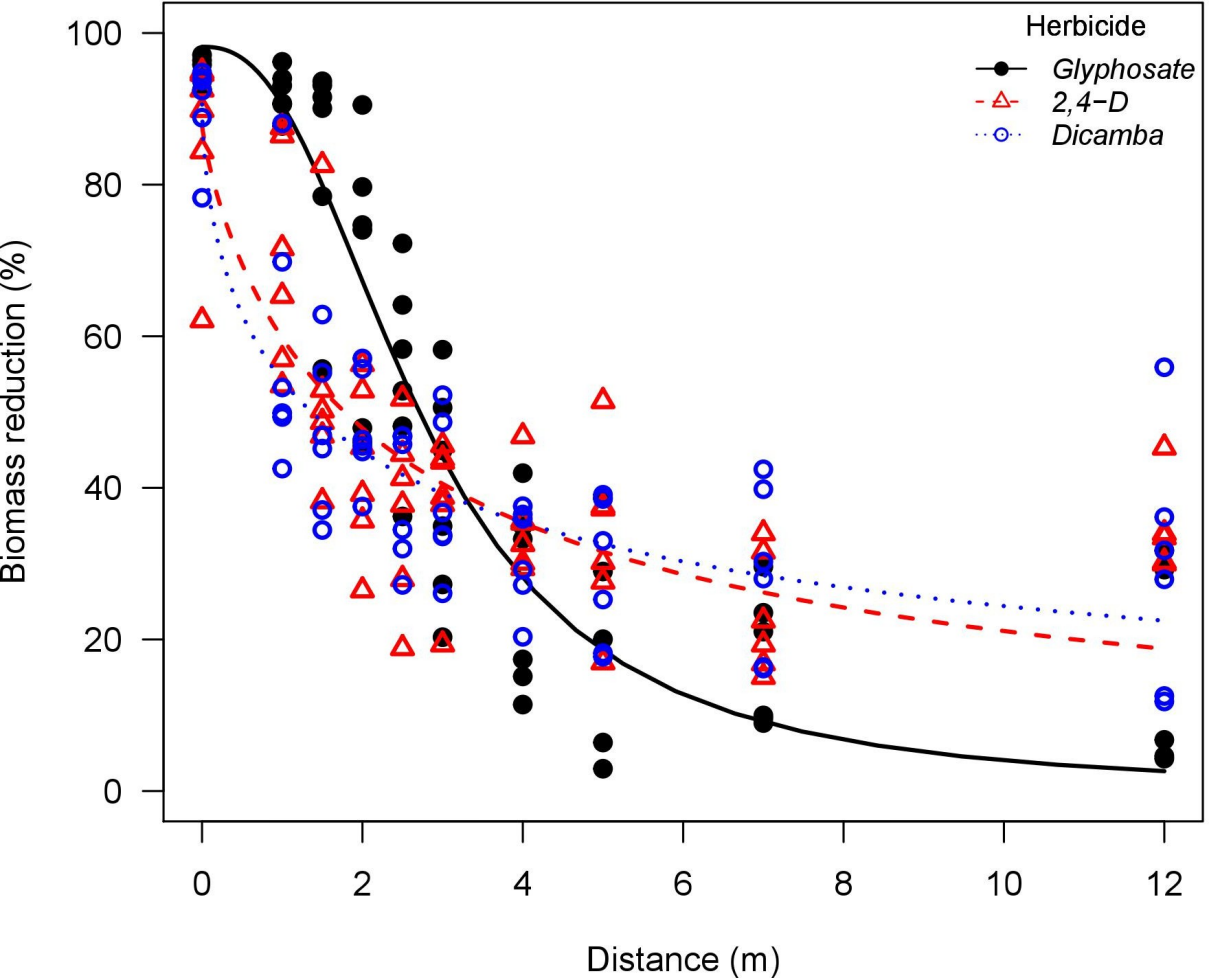
Waterhemp (*Amaranthus tuberculatus*) biomass reduction as influenced by glyphosate, 2,4-D, and dicamba particle drift using an air-inclusion nozzle (AI95015EVS) in a low speed wind tunnel. Glyphosate solution had the addition of ammonium sulfate solution at 5% v/v (Bronc^®^, Wilbur-Ellis Agribusiness, Aurora, CO, USA).

**Fig 8:**
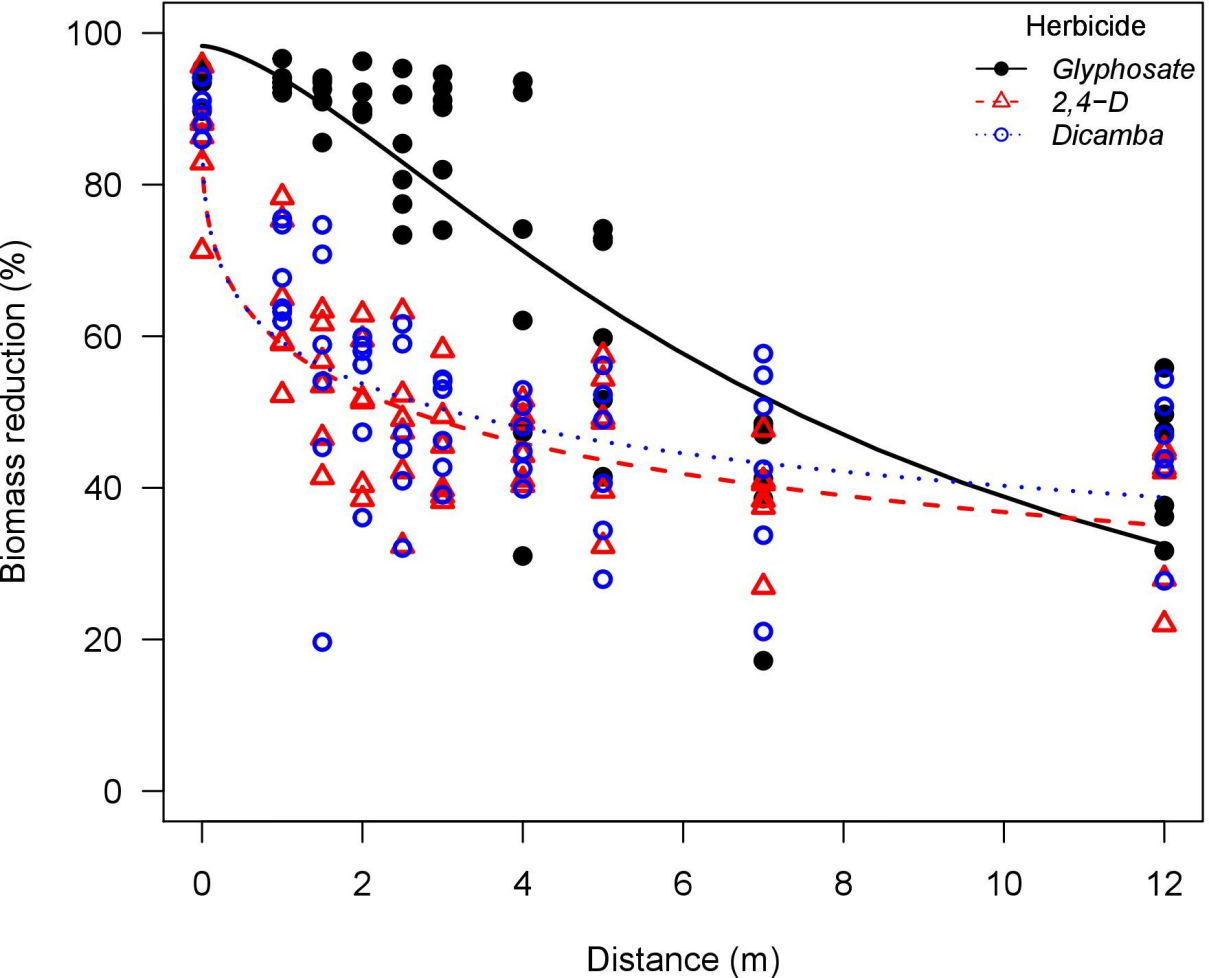
Palmer amaranth (*Amaranthus palmeri*) biomass reduction as influenced by glyphosate, 2,4-D, and dicamba particle drift using an air-inclusion nozzle (AI95015EVS) in a low speed wind tunnel. Glyphosate solution had the addition of ammonium sulfate solution at 5% v/v (Bronc^®^, Wilbur-Ellis Agribusiness, Aurora, CO, USA).

Glyphosate was more active at higher exposure rates compared to 2,4-D and dicamba. The *e* parameters (distance to 50% biomass reduction) also support this observation. Glyphosate applications had greater *e* parameter when compared to 2,4-D and dicamba, especially in applications with the flat-fan nozzle where plants are exposed to higher herbicide rates. Conversely, 2,4-D and dicamba were more active than glyphosate under lower exposure rates. This is more evident in the biomass reduction curves for waterhemp and Palmer amaranth exposed to herbicide drift from the AI nozzle (Figs 7 and 8). In fact, glyphosate applications had greater *b* parameter (slope at the inflection point) in general, indicating that biomass reduction curves had faster decay rate as the downwind distance was increased when compared to 2,4-D and dicamba. This indicates that glyphosate would reach no observable effect level at shorter downwind distances when compared to 2,4-D and dicamba. This corroborates previous reports relating low rates of 2,4-D and dicamba to high crop injury potential on soybean (*Glycine max* (L.) Merr.), cotton (*Gossypium hirsutum* L.), tomato (*Solanum lycopersicum* L.), and other broadleaf species [48–50].

### Herbicide drift and plant exposure to sublethal rates

Estimations of spray drift deposition as influenced by downwind distance and nozzle type (pooled across herbicides) are provided in Table 7. Applications with the flat-fan nozzle resulted in near-field spray drift ranging from 32.3 (1.0 m) to 11.5% (3.0 m) of the applied rate. The use of the AI nozzle decreased the dose exposure in the same distance range, with drift deposition estimations ranging from 11.4 (1.0 m) to 2.7% (3 m) of the applied rate.

**Table 7:** Spray drift deposition estimations with 95% confidence intervals (CI 95%) as influenced by downwind distance and nozzle type (pooled herbicides) using a log-logistic non-linear regression model in the spray particle drift study.

Nozzle^a^	Distance	Spray deposition^b^	CI 95%
	m	^_________________________^ % ^_________________________^	
TP95015EVS	1.0	32.3	(31.1–33.5)
	1.5	22.8	(22.1–23.5)
	2.0	17.4	(16.8–18.0)
	2.5	13.9	(13.3–14.5)
	3.0	11.5	(10.9–12.1)
	4.0	8.5	(7.9–9.1)
	5.0	6.7	(6.1–7.2)
	7.0	4.6	(4.1–5.1)
	12.0	2.5	(2.1–2.8)
AI95015EVS	1.0	11.4	(10.1–12.8)
	1.5	6.8	(6.1–7.5)
	2.0	4.7	(4.0–5.3)
	2.5	3.4	(2.8–4.1)
	3.0	2.7	(2.1–3.3)
	4.0	1.8	(1.3–2.4)
	5.0	1.3	(0.9–1.8)
	7.0	0.8	(0.5–1.2)
	12.0	0.4	(0.2–0.6)

^a^TeeJet Technologies, Spraying Systems Co., Glendale Heights, IL.

^b^Spray drift deposition (%) in relation to the applied rate of 140.3 L ha^-1^.

It has been reported that progenies of an initially susceptible population of annual ryegrass (*Lolium rigidum* Gaudin) shifted towards glyphosate resistance (up to 2.1-fold in the LD_50_) after being recurrently selected with sublethal rates of glyphosate [21]. These authors exposed three generations of *Lolium rigidum* plants to sublethal rates of glyphosate ranging from 150 g ae ha^-1^ to 350 g ae ha^-1^ (17 to 40% of the 867 g ae ha^-1^ commonly adopted field rate in glyphosate tolerant crops). In a similar study, it was reported that a glyphosate-susceptible Palmer amaranth population evolved glyphosate resistance (2.2-fold in the LD_50_) after being recurrently selected under sublethal rates of glyphosate for four generations [25]. The author reported that glyphosate doses of 105, 126, 210, and 420 g ae ha^-1^ (12, 15, 24, and 48% of the 867 g ae ha^-1^ commonly adopted field rate in glyphosate tolerant crops_,_ respectively) were used as generations progressed during the recurrent selection study. In a *Raphanus raphanistrum* L. population, the plants evolved 2,4-D resistance (8.6-fold in the LD_50_) after being recurrently selected during four generations [18]. The authors exposed plants to 125, 250, and 750 g 2,4-D ae ha^-1^ (12, 24, and 73% of the 1065 g ae ha^-1^ recommended rate for 2,4-D-tolerant soybean) as generations progressed. Another study reported that a 2,4-D and dicamba-susceptible Palmer amaranth population had its susceptibility reduced to both herbicides (2.8 and 2.0-fold in the LD_50_ for dicamba and 2,4-D, respectively) after recurrent selection with sublethal rates of dicamba for three selection generations [26]. The authors exposed plants to 140, 280, and 420 g dicamba ae ha^-1^ (25, 50, and 75% of the 560 g ae ha^-1^ recommended rate for dicamba-tolerant soybean) during the selection generations. Recurrent selection studies with sublethal rates of pyroxasulfone and diclofop-methyl were also associated with resistance evolution in weeds in previous studies [19,20,22,23,27].

Despite similar dose ranges, herbicide drift exposure differs from previously reported sublethal rate studies in terms of spray deposition pattern on plants and herbicide concentration within spray droplets. Unlike an intentional sublethal rate application with a constant carrier volume (usually ranging from 94 to 188 L ha^-1^), spray drift deposition is not consistent across field edges, which could influence plant response to the herbicide exposure. The higher herbicide concentration of spray drift droplets at lower carrier volumes could also influence plant response to herbicide exposure. Previous research indicated that glyphosate was more active at lower carrier volumes (more concentrated droplets) on oat (*Avena sativa* L.), wheat (*Triticum aestivum* L.), and several annual grass weed species such as *Echinochloa crusgalli* L., *Panicum dichotomiflorum* Michx., *Setaria viridis* (L.) Beauv., *Setaria pumila* (Poir.) Roem. et Schult, and *Digitaria sanguinalis* (L.) Scop., especially when lower glyphosate rates were compared [51,52]. Another study reported that carrier volume also influenced glyphosate activity on corn, whereas soybean was not affected [53,54]. It has also been reported that carrier volume influenced low rates of 2,4-D activity on cotton plants with lower carrier volumes (more concentrated droplets) resulting in more herbicide injury [53]. Similarly, lower rates of 2,4-D and dicamba were more active on cotton when lower carrier volumes with more concentrated droplets were used [50]. A study highlighted that the active ingredient concentration within droplets could influence the diffusion process of herbicide foliar uptake [55]. However, the authors mentioned that glyphosate foliar uptake has been investigated more than other herbicides. Additionally, it has been suggested that carrier volume could influence glyphosate activity because of water hardness, surfactant concentration, and spray droplet dynamics [52]. Herbicides tested in this study (glyphosate, 2,4-D, and dicamba) have systemic activity and can still be effective at lower carrier volumes and coverage, whereas contact herbicides usually require higher carrier volumes and adequate coverage [56–58]. Therefore, spray drift and injury potential from contact herbicides needs to be further investigated.

The results of this study indicate that herbicide drift towards field edges expose weeds to a range of herbicide rates reported to select for herbicide resistance. A previous study reported that only 3% of a total of 215 Palmer amaranth populations collected from roadsides, ditches, and field borders in eastern Arkansas were completely susceptible to glyphosate [14]. Glyphosate resistance was also confirmed in waterhemp and Palmer amaranth populations located on field borders and ditches in Nebraska [17]. Similarly, the presence of herbicide-resistant giant ragweed (*Ambrosia trifida* L.) in crop fields throughout the U.S. Corn Belt and Ontario (Canada) was strongly correlated to the species presence on crop field edges such as railroad sidings, ditch banks, and fencerows [15].

This study confirmed that nozzle selection influenced spray drift and consequent herbicide dose exposure on field edges, although spray drift could also be influenced by other parameters not tested, such as wind speed and boom height. The distance range with herbicide exposure and selection pressure is further increased for applications with the flat-fan nozzle (higher drift potential). It has been suggested that plants exposed to low doses of herbicides experience physiological stress, whereas plants exposed to even lower rates (hormetic doses) could also be subjected to stress [34]. Therefore, further studies are necessary to investigate if weeds could evolve herbicide resistance after recurrent selection with different exposure ranges of herbicide drift.

Despite the herbicide drift exposure and its potential implications on resistance evolution and weed management, near-field weed populations are often neglected and not properly managed in agricultural landscapes [14,15,17,37]. It has been reported that unmanaged field margins with resistant-prone weeds can exacerbate the risk of resistance, especially when outcrossing occurs with resistant populations near field [37]. Having plants under selection pressure for herbicide resistance on field borders could be detrimental for in-field weed management as pollen-mediated gene flow plays an important role in dispersing herbicide resistance alleles in cross-pollinated species such as waterhemp and Palmer amaranth [59–61]. Preventing resistance-prone weeds on field margins is an important best management practice (BMP) to delay herbicide resistance, although the additional management costs and time constraints pose a challenge for growers [18,62]. Growers should consider additional strategies to mitigate near-field spray drift [43,63], and implement appropriate control strategies to manage weed populations on field borders, such as mowing, using boomless nozzles for weed control in areas of difficult access (fencerows, electrical lines), or planting and maintaining field borders to a less-weedy and easier to manage species [37].

## Supporting information

S1 File Study data. (ZIP)
